# Synthesis of Terpineol from Alpha-Pinene Catalyzed by *α*-Hydroxy Acids

**DOI:** 10.3390/molecules27031126

**Published:** 2022-02-08

**Authors:** Zhong-Lei Meng, Ru-Si Wen, Xiao-Rui Huang, Rong-Xiu Qin, Yi-Ming Hu, Yong-Hong Zhou

**Affiliations:** 1 Institute of Chemical Industry of Forest Products, CAF, Nanjing 210042, China; meng-zhonglei@163.com; 2 Guangxi Key Laboratory of Superior Timber Trees Resource Cultivation, Guangxi Forestry Research Institute, Nanning 530002, China; luse-rose@163.com (R.-S.W.); hxr201400810091@yahoo.com (X.-R.H.); qrx20151112@126.com (R.-X.Q.); 3 School of Pharmacy, Guizhou University of Traditional Chinese Medicine, Guiyang 550025, China; huyiminglysh@126.com

**Keywords:** pinene, terpineol, alpha-hydroxy acids, phosphoric acid, synthesis

## Abstract

We report the use of five alpha-hydroxy acids (citric, tartaric, mandelic, lactic and glycolic acids) as catalysts in the synthesis of terpineol from alpha-pinene. The study found that the hydration rate of pinene was slow when only catalyzed by alpha-hydroxyl acids. Ternary composite catalysts, composed of AHAs, phosphoric acid, and acetic acid, had a good catalytic performance. The reaction step was hydrolysis of the intermediate terpinyl acetate, which yielded terpineol. The optimal reaction conditions were as follows: alpha-pinene, acetic acid, water, citric acid, and phosphoric acid, at a mass ratio of 1:2.5:1:(0.1–0.05):0.05, a reaction temperature of 70 °C, and a reaction time of 12–15 h. The conversion of alpha-pinene was 96%, the content of alpha-terpineol was 46.9%, and the selectivity of alpha-terpineol was 48.1%. In addition, the catalytic performance of monolayer graphene oxide and its composite catalyst with citric acid was studied, with acetic acid used as an additive.

## 1. Introduction

Terpineol has an aroma similar to lilac. It is present in a variety of essential oils and is widely used in the fragrance industry. In addition to its traditional use, terpineol has been extensively exploited by the pharmaceutical industry, due to its antioxidant, anti-inflammatory, anti-proliferative, anti-microbial, and analgesic effects [1].

As early as 1947, Mosher studied alpha-pinene hydration products catalyzed by acids. The industrial hydration of turpentine oil is typically catalyzed by sulfuric acid, which produces large amounts of solid/liquid/gas waste, causing serious environmental pollution and complicating downstream processes [2]. To address the strong corrosion in alpha-terpineol synthesis when sulfuric acid is used as a catalyst, Román-Aguirre et al. used hydrochloric acid, acetic acid, oxalic acid, and monochloroacetic acid (MCA) as catalysts for the direct synthesis of terpineol from alpha-pinene [3]. The study showed that MCA was the most effective catalyst, with a 90% conversion of alpha-pinene; the selectivity of terpineol reached 65%.

Solid acids and ionic liquids as catalysts have been intensively studied because they can be easily separated from the products. However, chloroacetic acids are needed as promoters. Yang et al. prepared acid-doped nanomaterials by rapidly mixing polyaniline nanofibers with hydrochloric, sulfuric, and MCA as catalysts for terpineol synthesis [4]. Yu et al. used solid superacid SO_4_^2−^/SnO_2_, composite solid superacid SO_4_^2−^/SiO_2_-ZrO_2_, and mesoporous zirconium-based molecular sieve SO_4_^2−^/Zr-MCM-41 compounds as catalysts; MCA was used as an esterification agent for one-step synthesis of terpineol from turpentine [5,6,7]. Chen et al. used solid superacid Ni/SO_4_^2−^-SnO_2_ as a catalyst and MCA as the promoter to synthesize alpha-terpineol from alpha-pinene [8]. Wang et al. produced terpineol through the hydration of turpentine with solid superacid MoO_3_/ZrO_2_ as the catalyst and MCA as the promoter [9]. Yang et al. compared the effects of three esterification agents (glacial acetic acid, MCA, and trichloroacetic acid (TCA)) for the synthesis of terpineol from turpentine, catalyzed by solid superacid SO_4_^2−^/ZrO_2_. These researchers concluded that MCA was the most effective [10]. Liu et al. used a thermoregulated acidic ionic liquid, 1-(3-sulfonic acid)-propyl-3-polyethylene glycol imidazole dihydrogen phosphate [PEOIM-SO_3_H]H_2_PO_4_, as the catalyst and MCA as the promoter for the hydration of alpha-pinene to terpineol [11]. Chen et al. prepared a carbon–silicon composite solid acid, via carbonization and sulfonation as the catalyst, and used MCA as the promoter for the hydration of alpha-pinene to terpineol [12]. Wijayati et al. prepared a catalyst by impregnating TCA on a Y-zeolite molecular sieve for the synthesis of terpineol from alpha-pinene [13]. Wijayati et al. also prepared a TCA/ZHY catalyst (Si/Al = 3.25) via impregnation to achieve terpineol synthesis [14]. Ávila et al. prepared catalysts for pinene hydration by impregnating TCA on SiO_2_, TiO_2_, and ZrO_2_·nH_2_O [15]. Sekerová et al. treated montmorillonite K10 with several acids (H_2_SO_4_, HCl, HNO_3_, and MCA) to catalyze the hydration of alpha-pinene [16]. Comelli et al. prepared a catalyst for alpha-pinene hydration by impregnating natural clay with MCA [17].

Solid acids without chloroacetic acids (MCA or TCA) as the promoter require large amounts of acetone or isopropanol as solvents to facilitate the production of terpineol from alpha-pinene [18,19,20]. Chloroacetic acids are highly corrosive and harmful to humans (TCA is a class 2B carcinogen) and, thus, it is important to find non-toxic and environmentally friendly organic acids, with similar catalytic activity as chloroacetic acids (MCA or TCA) for alpha-pinene hydration.

Most alpha-hydroxy acids (AHAs) are widely distributed in nature. They are environmentally friendly, non-toxic to humans and animals, and mildly corrosive toward reaction equipment. Here, we used common AHAs (citric acid, L-(+)-tartaric acid, DL-mandelic, L-(+)-lactic acid, and glycolic acid) as catalysts for alpha-pinene hydration [21,22,23] and then investigated and compared their catalytic performance and reaction steps.

## 2. Results and Discussion

### 2.1. Catalytic Performance of AHAs for Alpha-Pinene Hydration

**Figure 1:**
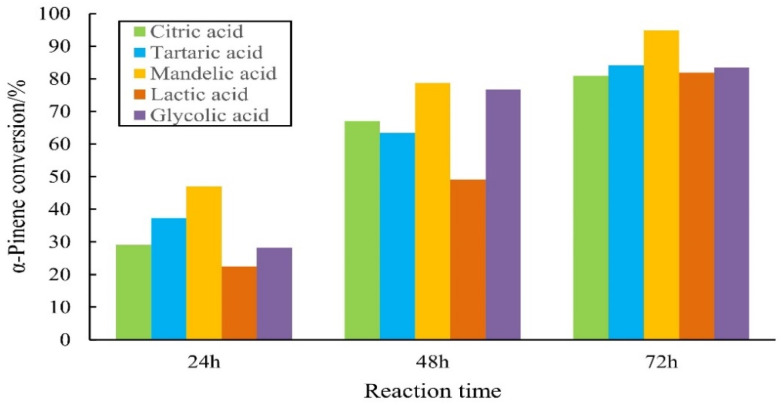
Comparison of conversion of alpha-pinene with AHA as the catalyst. Note: the mass ratio of alpha-pinene, water, and AHA was 1:1:1, at 70 °C.

**Figure 2:**
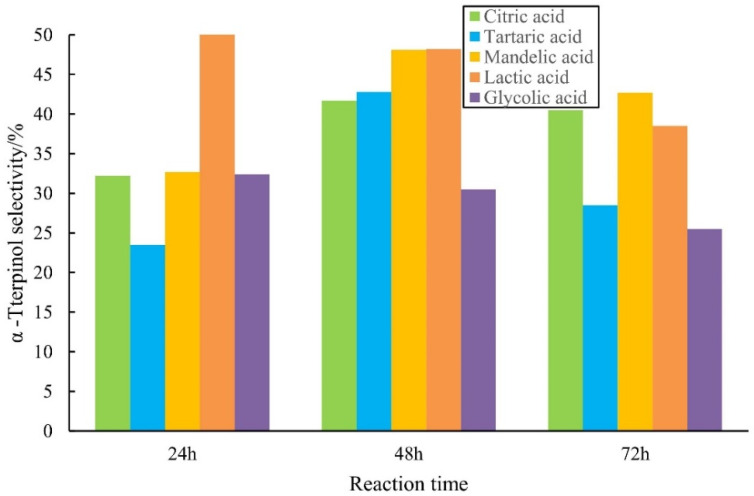
Selectivity comparison of alpha-terpinol with AHA as the catalyst. Note: The mass ratio of alpha-pinene, water and AHA was 1:1:1 at 70 °C.

**Figure 3:**
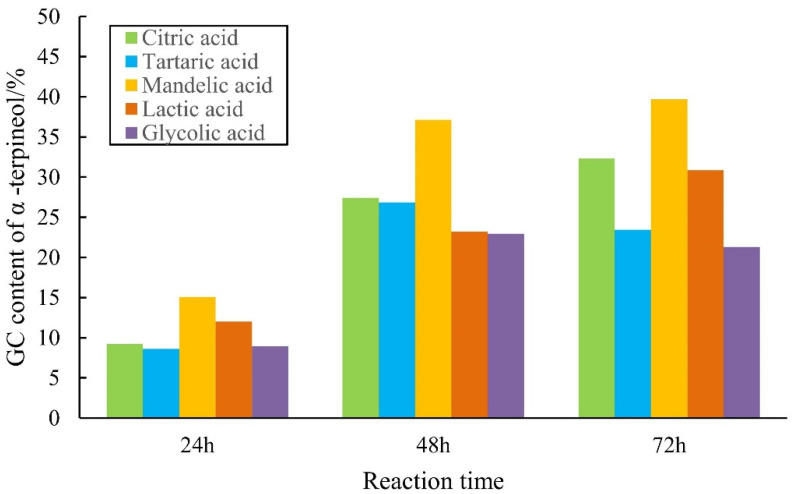
Comparison of alpha-terpineol content with AHA as the catalyst. Note: The mass ratio of alpha-pinene, water, and AHA was 1:1:1 at 70 °C.

Alpha-pinene hydration was catalyzed by five AHAs (citric acid, L-(+)-tartaric acid, DL-mandelic, L-(+)-lactic acid, and glycolic acid). The results are shown in Figure 1, Figure 2 and Figure 3. Figure 1 shows that the highest conversion of alpha-pinene was 94.8% at 72 h, and the final conversion of alpha-pinene followed the order of DL-mandelic > L-(+)-tartaric acid > citric acid > glycolic acid > L-(+)-lactic acid.

Figure 2 shows that the selectivity of alpha-pinitol increased and then decreased as the reaction progressed (except for L-(+)-lactic acid). Citric and DL-mandelic acid showed > 40% selectivity after 72 h. The final terpineol selectivity followed the order of DL-mandelic > citric acid > L-(+)-lactic acid > L-(+)-tartaric acid > glycolic acid.

Figure 3 shows that the highest content of alpha-terpineol in the product was 39.7%, and the content of alpha-terpineol in the final product followed the order of DL-mandelic > citric acid > L-(+)-lactic acid > L-(+)-tartaric acid > glycolic acid.

In the absence of a promoter, the hydration reaction catalyzed by AHAs was slow, due to the polarity differences between alpha-pinene and water, thus resulting in high resistance to mass transfer between the oil and water phases. As a result, promoters that promote immiscibility between oil and water should be added to accelerate the reaction.

### 2.2. Catalytic Performance of AHAs with Acetic Acid as the Promoter

**Figure 4:**
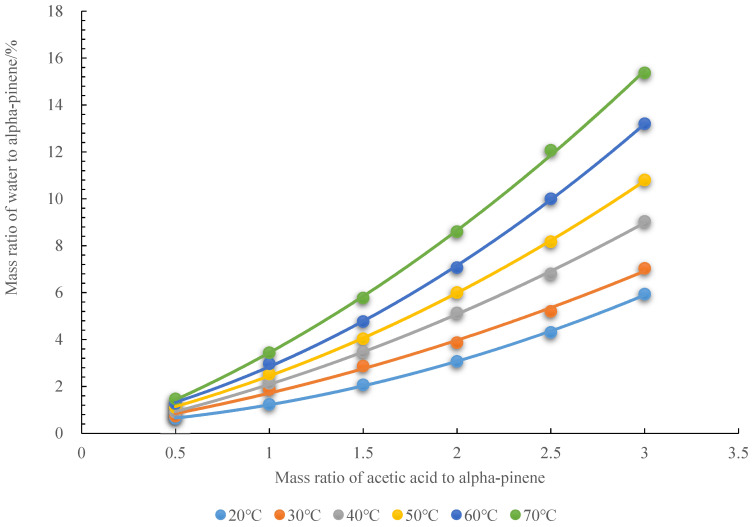
Acetic acid promotes the dissolution of water in alpha-pinene.

To improve the hydration of alpha-pinene, the protic liquid acetic acid was used as a promoter. Figure 4 shows that the solubility of water in alpha-pinene increased with increasing mass ratio of acetic acid to alpha-pinene. It also increased with temperature. When the molar ratio of water to alpha-pinene is 1:1, and the temperature is 70 °C, the minimum amount of acetic acid to maintain a single phase in the system can be obtained by solving experimental Equation (6), shown in Figure 4.

(6)y_(70°C)_ = 0.8x^2^ + 2.8133x − 0.1733

(18 ÷ 136) × 100 = 0.8x^2^ + 2.8133x − 0.1733

x ≈ 2.7

when the molar ratio of water to alpha-pinene is 1:1, the mass ratio of acetic acid to alpha-pinene is about 2.7.

y_(20__°C)_= 0.4833x^2^ + 0.4055x + 0.33 (1)

y_(30__°C)_ = 0.3452x^2^ + 1.2202x + 0.15 (2)

y_(40__°C)_ = 0.4524x^2^ + 1.6357x − 4 × 10^−15^ (3)

y_(50__°C)_ = 0.619x^2^ + 1.6829x + 0.1467 (4)

y_(60__°C)_ = 0.8595x^2^ + 1.7383x + 0.2433 (5)

y_(70__°C)_ = 0.8x^2^ + 2.8133x − 0.1733 (6)

Table 1 shows that the highest alpha-pinene conversion was 95.2% at 15 h, suggesting that acetic acid accelerated alpha-pinene hydration.

The citric acid content was reduced to 10% of the value shown in Table 1 and the amount of acetic acid increased four-fold (Table 2). As shown in Table 1 and Table 2, increasing the acetic acid content did not effectively increase the alpha-pinene hydration rate when the AHA content was reduced.

### 2.3. Effect of Phosphoric Acid on Alpha-Pinene Hydration Catalyzed by AHAs

The hydration of alpha-pinene was slow with phosphoric acid (Table 3), in the absence of acetic acid as a promoter. After 24 h, the conversion of alpha-pinene reached its maximum of 19.8%, and the highest alpha-terpineol selectivity (58.8%) was achieved by the catalyst composed of citric and phosphoric acids.

The hydration of alpha-pinene was promoted by acetic acid (Table 4). The conversion of alpha-pinene reached 99.5% and the selectivity of alpha-terpineol reached 48% after reacting for 15 h.

These experiments show that the combination of an AHA, phosphoric acid, and acetic acid could better catalyze the hydration reaction of alpha-pinene than a catalyst containing only one or two of these acids. The effects of citric acid, phosphoric acid, and acetic acid on alpha-pinene hydration were studied in detail through the following single-factor and orthogonal experiments. The hydration process of alpha-pinene catalyzed by citric acid, phosphoric acid, and acetic acid is also discussed.

### 2.4. Single-Factor Experiments on Alpha-Pinene Hydration

**Figure 5:**
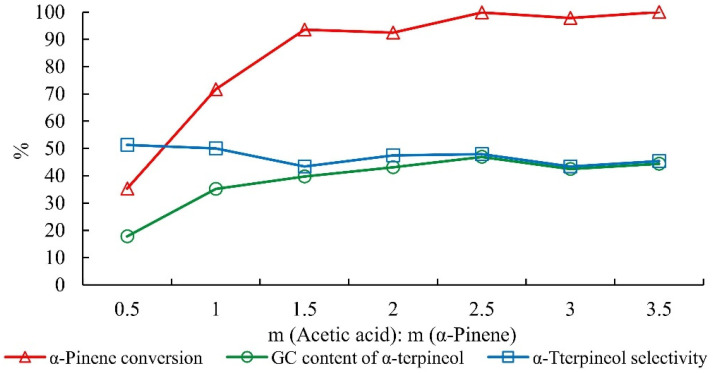
Effect of acetic acid dosage on the hydration of alpha-pinene. Note: The mass ratio of alpha-pinene, water, AHA, and phosphoric acid was 1:1:0.1:0.05 for 15 h.

**Figure 6:**
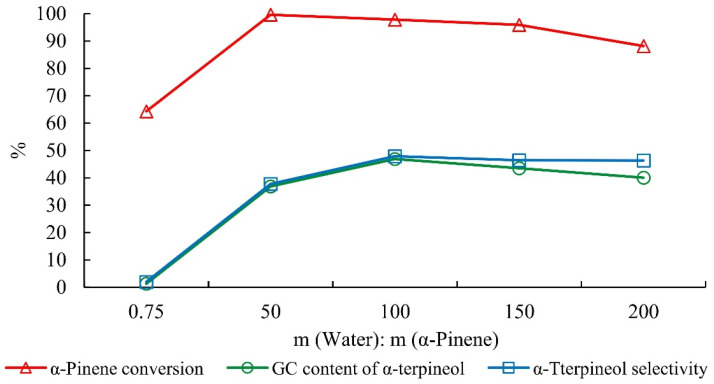
Effect of water dosage on the hydration of alpha-pinene. Note: The mass ratio of alpha-pinene, acetic acid, citric acid, and phosphoric acid was 1:2.5:0.1:0.05 for 15 h.

**Figure 7:**
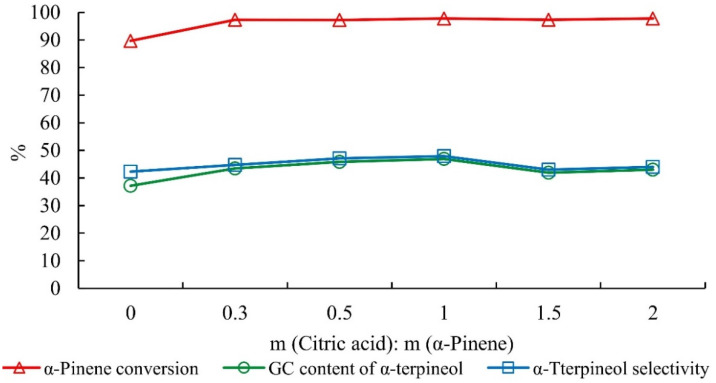
Influence of citric acid dosage on the hydration of alpha-pinene. Note: The mass ratio of alpha-pinene, water, acetic acid, and phosphoric acid was 1:1:2.5:0.05 for 5 h.

**Figure 8:**
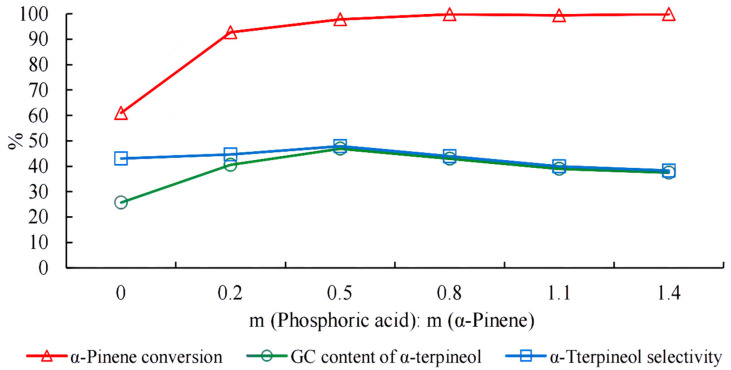
Effect of phosphoric acid dosage on the hydration of alpha-pinene. Note: The mass ratio of alpha-pinene, water, acetic acid, and citric acid was 1:1:2.5:1 for 15 h.

**Figure 9:**
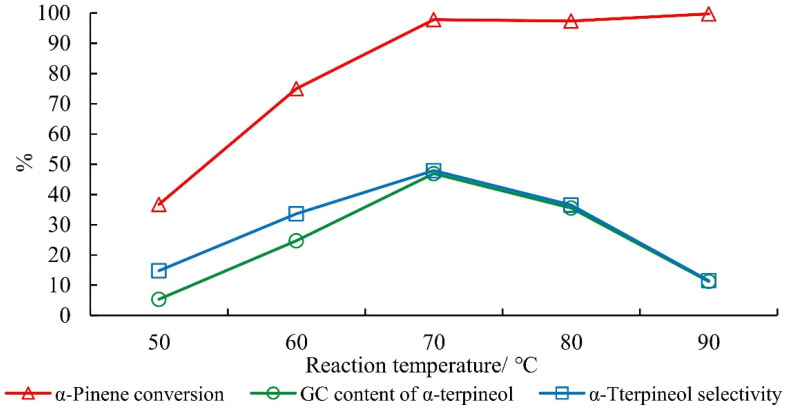
Effect of reaction temperature on the hydration of alpha-pinene. Note: Mass ratio of alpha-pinene, water, acetic acid, citric acid, and phosphoric acid was 1:1:2.5:0.1:0.05 for 15 h.

**Figure 10:**
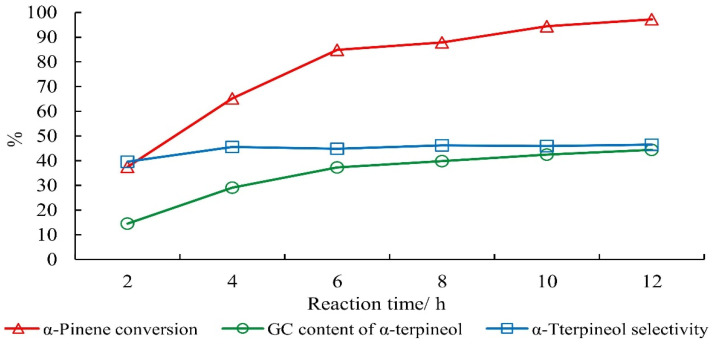
Effect of reaction temperature on the hydration of alpha-pinene. Note: The mass ratio of alpha-pinene, water, acetic acid, citric acid and phosphoric acid was 1:1:2.0.5:1:0.05 for 15 h.

We also investigated the effects of acetic acid, water, citric acid, phosphoric acid, reaction time, and the reaction temperature on alpha-pinene hydration (Figure 5, Figure 6, Figure 7, Figure 8, Figure 9 and Figure 10). The conversion of alpha-pinene and the content of alpha-terpineol in the product increased with increasing the mass ratio of acetic acid to alpha-pinene; the conversion of alpha-pinene was nearly 100% at mass ratios above 2.5. However, the selectivity of terpineol did not change significantly (Figure 5).

At a constant mass of the catalyst, the increase in water content reduced the concentration of the catalyst and the conversion of alpha-pinene (Figure 6). The water was quickly consumed by terpineol formation when a small amount of water was present (from the catalyst phosphoric acid). This increased the concentration of the catalyst in the reaction system and accelerated the isomerization of alpha-pinene, which in turn decreased alpha-terpineol selectivity.

The alpha-pinene conversion increased by ~8% with citric acid versus phosphoric acid alone (Figure 7). The content and selectivity of alpha-terpineol also increased and then decreased slightly with an increase in citric acid. Versus phosphoric acid alone, the content of alpha-terpineol increased by 9.7%, and the selectivity increased by 5.6% with citric acid.

Under experimental conditions, the conversion of alpha-pinene increased with an increase in phosphoric acid, although the increase was insignificant when the mass ratio of phosphoric acid to alpha-pinene was greater than 0.5 (Figure 8). The content and selectivity of alpha-terpineol increased and then decreased with phosphoric acid concentration. It reached a maximum with a phosphoric acid/alpha-pinene mass ratio of 0.5. Excess phosphoric acid promoted the isomerization of alpha-pinene to limonene and terpinolene, which led to a decrease in the alpha-terpineol content and product selectivity.

The conversion of alpha-pinene increased with temperature, but the selectivity of terpineol decreased rapidly at temperatures above 80 °C (Figure 9). This was mainly because phosphoric acid easily isomerized alpha-pinene to limonene and terpinolene at high temperatures.

The conversion of alpha-pinene and the selectivity of terpineol increased during the initial stages of the reaction, and the conversion of alpha-pinene was nearly complete (97.2%) after 12 h (Figure 10).

### 2.5. Orthogonal Experiment on Alpha-Pinene Hydration

Table 5 shows that the R values for the direct analysis of alpha-pinene conversion followed the order of A > B > F > E > C > D. The top three factors that affected alpha-pinene conversion were acetic acid, water, and temperature. Thus, the optimum reaction conditions were alpha-pinene, water, acetic acid, and citric acid, at a mass ratio of 1:3:0.5:0.1:0.05, a reaction temperature of 80 °C, and a reaction time of 15 h (A_3_B_1_C_2_D_2_E_3_F_3_).

Table 5 shows the results of the orthogonal experiment on alpha-terpineol selectivity. The R values for direct analysis were F > C > B > A > E > D. The top three factors that affected the selectivity of alpha-terpineol were temperature, citric acid, and water. Thus, the optimum reaction conditions were alpha-pinene, water, acetic acid, and citric acid, at a mass ratio of 1:1:1.5:0.05:0.05, a reaction temperature of 70 °C, and a reaction time of 10 h (A_1_B_3_C_1_D_1_E_2_F_2_).

Using the conversion of alpha-pinene and the selectivity of alpha-terpineol as responses, the orthogonal and single-factor experiments suggested that the optimum alpha-pinene hydration conditions were alpha-pinene, acetic acid, water, citric acid, and phosphoric acid, at a mass ratio of 1:2.5:1:(0.1–0.05):0.05, a reaction temperature of 70 °C, and a reaction time of 12–15 h.

### 2.6. Reaction Pathway for the Hydration of Alpha-Pinene

**Figure 11:**
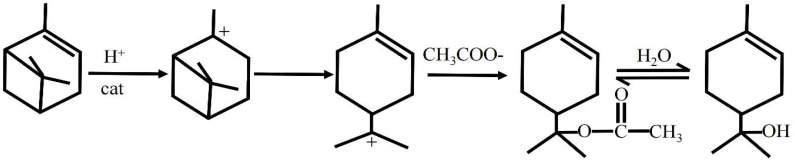
The main stages of the target reaction.

The process of alpha-pinene hydration using acetic acid as the promoter and AHA plus phosphoric acid as the catalyst is shown in Figure 11.

Yang proposed that acetic acid and chloroacetic acid could serve as esterification agents when a solid superacid was used as the catalyst for alpha-pinene hydration; terpinyl chloroacetate formed as the intermediate, which was then hydrolyzed to yield terpineol [10]. However, neither Yang nor Yu provided evidence for this stage [5,10]. Therefore, a comparative experiment was designed to test the presence of this reaction stage in this study.

**Figure 12:**
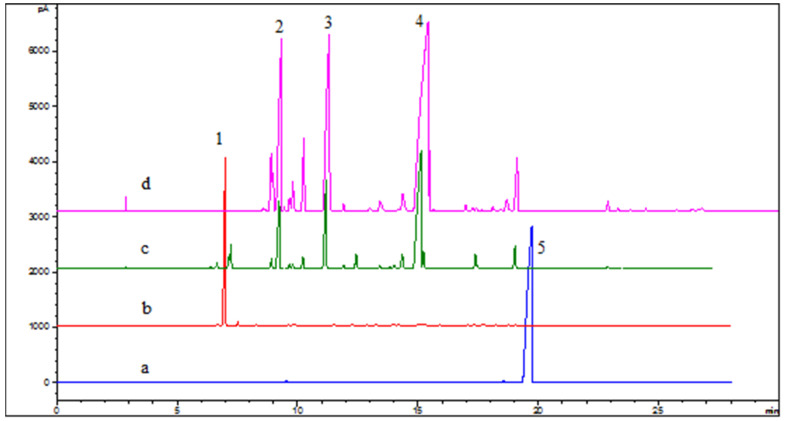
Gas chromatogram of raw materials and products, **a**. terpinyl acetate; **b**. alpha-pinene; **c**. hydration products of alpha-pinene; **d**. terpinyl acetate hydrolysate. Note: **1**. alpha-pinene; **2**. limonene; **3**. iso-terpinene; **4**. alpha-terpineol; **5**. terpinyl acetate.

The reaction conditions for the confirmation experiment were terpinyl acetate (or alpha-pinene), acetic acid, water, citric acid, and phosphoric acid, at a mass ratio of 1:2.5:1:0.05:0.05. With terpinyl acetate as the substrate, we performed experiments A (with acetic acid) and B (without acetic acid). We also conducted experiments C (with added water) and D (with trace amounts of water from phosphoric acid), using alpha-pinene as the substrate (Table 6 and Figure 12).

Experiments A and C, with acetic acid as the promoter and citric acid plus phosphoric acid as the catalyst, showed similar product compositions. As shown in previous experiments, the reaction of alpha-pinene with water was very slow without the presence of acetic acid. The use of solvents, such as isopropyl alcohol and acetone for reactions in the homogeneous systems did not significantly promote terpineol formation. Therefore, these results suggested that acetic acid not only improved phase-to-phase mass transfer but also led to the formation of the reaction intermediate.

Table 6 shows that the hydrolysis of terpinyl acetate was very slow without acetic acid (experiment C); the conversion was only 8.7% after reacting for 15 h. With a trace amount of water (experiment D), alpha-pinene reacted with acetic acid and the conversion of alpha-pinene reached 95%. However, the amount of terpinyl acetate in the product was only 14.1%. This suggested that both acetic acid and water played important roles in the hydrolysis of terpinyl acetate and the hydration of alpha-pinene.

In addition, short-chain fatty acids were used as auxiliary agents, and the reaction conditions were as follows: the mass ratio of alpha-pinene, water, fatty acid, citric acid, and phosphoric acid was 1:1:2:0.05:0.1, the reaction temperature was 70 °C, and the reaction time was 20 h. Under these conditions, the order of alpha-pinene conversion was as follows: formic acid (100%) > acetic acid (98.6%) > propionic acid (86.1%) > butyric acid (28.3%) > isobutyric acid (12.2%). Although the pKa values of the short-chain fatty acids were similar, they all increased the miscibility of water and alpha-pinene. However, the conversion of alpha-pinene decreased as the carbon chain of the fatty acid molecular skeleton increased; the rate of the branched chain was lower than the straight chain. This also proved that when alpha-pinene synthesized terpineol, it passed through the ester intermediates. That is, fatty acids with a large steric hindrance had more difficulty forming esters, thus resulting in a low alpha-pinene conversion.

In general, racemic terpineol was obtained via dehydration of 1,8-terpene glycol, while terpineol synthesized via the AHA composite catalyst had a high optical rotation. Therefore, in contrast to the reaction process mentioned previously [24,25], the hydration of pinene catalyzed by AHA/phosphoric acid/acetic acid ternary composite catalyst probably does not go through 1,8-terpene glycol intermediate.

### 2.7. Comparative Experiments

Sulfuric acid, phosphoric acid, and one of the monolayer graphene oxides were used for a ternary composite catalyst with citric acid and acetic acid. The reaction of alpha-pinene was accelerated by the inorganic acids, and the selectivity of alpha-terpineol with the composite catalyst was higher than with inorganic acid alone (Table 7). Therefore, these findings suggested that the AHAs could stabilize the carbonium ions formed by alpha-pinene protonation, which was beneficial to the formation of the addition product.

**Figure 13:**
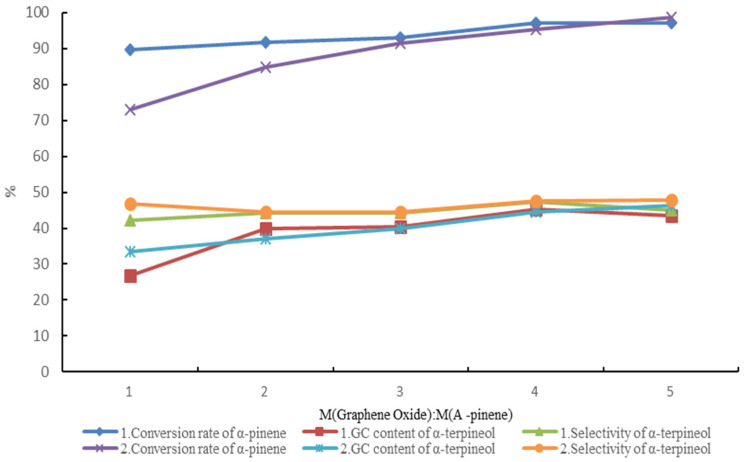
Hydration of alpha-pinene catalyzed by monolayer graphene oxide.

There are oxygen-containing groups on the surface and the edge of monolayer graphene oxide, e.g., epoxy, carboxyl, and hydroxyl groups [26]. The effects of monolayer graphene oxide and a citric acid composite catalyst (1) and monolayer graphene oxide catalyst (2) on the hydration of alpha-pinene were also studied. Figure 13 shows that the conversion of alpha-pinene and the GC content of terpineol in the product increased as the amount of monolayer graphene oxide increased; the selectivity of terpineol did not change much. Versus the monolayer graphene oxide catalyst (2), the composite catalyst (1) had higher alpha-pinene conversion and slightly lower terpineol selectivity. However, the reused monolayer graphene oxide had greatly reduced catalytic ability (the conversion of alpha-pinene was only 45%).

Additionally, trichloroacetic acid was also compared with citric acid under the same experimental conditions. Table 8 shows that citric acid as the catalyst increased the conversion of alpha-pinene by 6.6% compared with trichloroacetic acid. The content of alpha-pinene increased by 21.2%, and the selectivity of alpha-terpineol by 17%.

### 2.8. Infrared Spectrum, Optical Rotation, and Refractive Index of Alpha-Pinene Hydration Products

**Figure 14:**
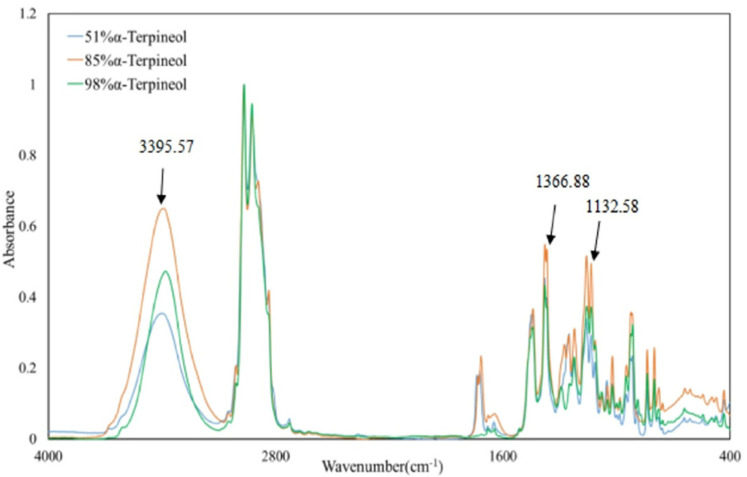
Infrared spectrum of terpineol sample.

Two samples of alpha-terpineol, with a GC content of 51% and 85%, were obtained from the hydration product of alpha-pinene. Reference terpineol (Macklin, Shanghai, China) with a GC content of 98% was also analyzed by infrared spectroscopy. Absorption is seen at 3395.57 cm^−1^, 1366.88 cm^−1^, and 1132.58 cm^−1^, corresponding to the OH stretching vibration, the in-plane deformation vibration of tertiary alcohol δ_OH_, and the stretching vibration of tertiary alcohol ν_C-O_, respectively (Figure 14). At 3395.57 cm^−1^, the peak shape is broad due to the hydrogen bond association between terpineol molecules.

The three terpineol samples had a GC content of 51%, 85%, and 98%. We determined their optical rotation, enantiomeric excess (ee), and refractive index. The optical rotations of the three samples were as follows: −45.3° (20 °C), −66.7° (20 °C), and 1.9° (20 °C), respectively. The ee values of the three samples were 71%, 65%, and 17.2%, respectively. The refractive indices of the three terpineol samples were 1.4818 (20 °C), 1.4782 (20 °C), and 1.4838 (21 °C), respectively. The alpha-terpineol synthesized in this experiment has higher optical rotation than the commercial reference alpha-terpineol (98%).

## 3. Materials and Methods

### 3.1. Materials and Instruments

The raw materials and reagents used in this study were (−)-alpha-pinene, 98% (Aladdin, Shanghai, China), terpinyl acetate, 95% (Aladdin), citric acid·H_2_O (Chron Chemicals, Chengdu, China), L-(+)-tartaric acid, 99% (Macklin, Shanghai, China), DL-mandelic acid, 99% (Macklin), L-(+)-lactic acid, 85%–90% (Chron Chemicals), glycolic acid, 98% (Aladdin), monolayer graphene oxide powder, ~65% carbon (Macklin), phosphoric acid (85%) and sulfuric acid (98%) (Xilong Scientific, Chengdu, China), acetic acid, 99.5% (Chron Chemicals), and distilled water was made in house.

The reaction vessel consisted of an organic synthesizer PPV-3000, Eyela, Tokyo, Japan. The analytical instruments consisted of a 7890A gas chromatograph (GC) (Agilent, Santa Clara, CA, USA), an AT-35 quartz capillary column (60 m × 0.25 mm × 0.25 μm), and a HP-CHIRAL-20B (30 m × 0.25 mm × 0.25 μm). The TQ456 gas chromatograph was coupled with a mass spectrometer (GC-MS) (Bruker, Billerica, MA, USA), and a BR-5 elastic quartz capillary column (30 m × 0.25 mm × 0.25 μm). The instrument used to measure the optical rotation was an SWG-2 automatic polarimeter with a test tube 100 mm long, and a 589-nm light source (Shanghai Precision Scientific Instrument Co., Ltd., Shanghai, China). The instrument for measuring refractive index was a WAY-2W Abbe refractometer (Shanghai Optical Instrument Factory, Shanghai, China). The infrared spectrometer was Thermo Nicolet iS50 Fourier Transform Infrared Spectrometer (Thermo Fisher Scientific, Waltham, MA, USA).

### 3.2. Experimental Methods

A flask was filled with 10 g of alpha-pinene, 25 g of acetic acid, 1 g of catalyst, and 10 g of water. This was magnetically stirred at 500 rpm, and the reaction was performed at 60–80 °C for 5–15 h. Upon completion, the product was transferred into a separatory funnel to separate the product (upper phase) from the acetic acid aqueous solution containing the catalyst (lower phase). The product phase was washed with water until it reached a neutral pH. The sample was then dried with anhydrous sodium sulfate and analyzed by GC or GC-MS. Based on the single-factor experiments, a six-factor, three-level orthogonal experiment was next designed to investigate the effects of various factors on alpha-pinene conversion and alpha-terpineol selectivity (Table 9).

### 3.3. Analytical Methods

The concentrations of raw materials and products were calculated by area normalization. According to the law of mass conservation, if the losses from evaporation and dissolution during the experiment are negligible, then the conversion of alpha-pinene can be approximated by subtracting the content of alpha-pinene in the product from its amount in the raw material, according to the following:

Alpha-pinene conversion = (content of alpha-pinene in raw material—content of alpha-pinene in the product)/content of alpha-pinene in the raw material.

Terpineol selectivity = content of alpha-terpineol in the product (content of alpha-pinene in the raw material—content of alpha-pinene in the product).

The GC conditions were as follows. The carrier gas consisted of high-purity nitrogen gas, and the oven temperature was 70 °C (2 min). This increased at 5 °C/min to 150 °C, held for 3 min, and 10 °C/min to 230 °C, and held for 2 min. The inlet temperature was 250 °C, the total flow rate was 130.5 mL/min, the split ratio was 50:1, and the septum purge flow rate was 2.5 mL/min; a flame ionization detector (FID) was used. The injection port temperature was 250 °C, the FID hydrogen flow rate was 40 mL/min, the FID air flow rate was 450 mL/min, the nitrogen gas flow rate was 25 mL/min, and the injection volume was 0.2 μL.

The GC-MS conditions were as follows. The carrier gas consisted of high-purity helium gas, and the oven temperature was 50 °C (3 min). The temperature increased at 20 °C/min to 120 °C, 2 °C/min to 180 °C (2 min), and at 50 °C/min to 250 °C. It was then held for 5 min. The inlet temperature was 230 °C, and the interface temperature was 250 °C.

The MS conditions included an EI ion source, an ionization voltage of 70 eV, and a scan range of 45–350 u. The full scan mode was set to solvent delay for 5 min, and the injection volume was 0.5 μL (sample was dissolved in ethanol at a mass fraction of 1%).

The alpha-terpineol samples were placed in an infrared spectrometer with air as the background value. The spectra were collected from 400 to 4000 cm^−1^ with a resolution of 4 cm^−1^ and 32 scans. We repeated the measurement 4 times for each sample, and the average value after deducting the background value was the infrared spectrum of the sample. The raw spectral data were calibrated using the calibration function that comes with OMNIC to remove the influence of the baseline. The analysis of the basic infrared spectra, peak fitting, and normalization were done by OMNIC Specta; SPSS 19.0 was used for other data normalization, and Excel was used for graphing.

## 4. Conclusions

(1) AHAs are environmentally friendly, non-toxic, and renewable organic acids that can catalyze the hydration of alpha-pinene. However, hydration with only AHAs was slow, and both acetic acid and inorganic acids were needed to accelerate the reaction. Phosphoric acid had a pKa similar to common AHAs (e.g., citric acid and L-(+)-tartaric acid), which increased the conversion of alpha-pinene and the selectivity of terpineol.
(2) During the hydration of alpha-pinene catalyzed by AHAs, acetic acid as the promoter enhanced the immiscibility between alpha-pinene and water. This led to the formation of intermediate terpinyl acetate. AHAs acted as stabilizers of carbonium ions, thus, driving the reaction in the direction of addition product formation.
(3) The optimal conditions for the hydration of alpha-pinene catalyzed by citric acid were alpha-pinene, acetic acid, water, citric acid, and phosphoric acid, at a mass ratio of 1:2.5:1:(0.1–0.05):0.05, a reaction temperature of 70 °C, and a reaction time of 12–15 h. The conversion of alpha-pinene was ≥96%, the content of alpha-terpineol was ≥46.9%, the selectivity of alpha-terpineol was ≥48.1%, and the yield was ≥ 85%.
(4) Monolayer graphene oxide could catalyze the hydration of alpha-pinene because it contained hydroxyl and carboxyl functional groups. However, monolayer graphene oxide was expensive and had poor reusability. This limited its value as a catalyst for alpha-pinene hydration reactions.

## Figures and Tables

**Table 1:** Comparison of catalytic performance of AHAs with acetic acid as the additive.

	Catalyst	Citric Acid	L-(+)-Tartaric Acid	DL-Mandelic	L-(+)-Lactic Acid	Glycolic Acid
Parmeter						
Alpha-pinene conversion (%)		75.7	95.2	76.5	62.0	82.3
GC content of alpha-terpineol (%)		26.9	22.7	25.2	26.5	29.9
Alpha-terpinol selectivity (%)		36.3	24.3	33.6	43.5	37.1

Note: The mass ratio of alpha-pinene, water, acetic acid, and AHA was 1:1:0.5:1 at 70 °C for 15 h.

**Table 2:** Catalytic effects of increasing the amount of acetic acid and decreasing the amount of citric acid.

	Reaction Time	24 h	48 h
Parmeter			
Alpha-pinene conversion (%)		72.8	74.9
GC content of alpha-terpineol (%)		31.7	34.5
Alpha-tterpinol selectivity (%)		43.6	46.1

Note: The mass ratio of alpha-pinene, water, acetic acid and citric acid was 1:1:2:0.1 at 70 °C.

**Table 3:** Effect of phosphoric acid on the hydration of alpha-pinene without acetic acid as the auxiliary agent.

	Catalyst	Citric Acid	L-(+)-Tartaric Acid	DL-Mandelic	L-(+)-Lactic Acid	Glycolic Acid
Parmeter						
Alpha-pinene conversion (%)		6.9	7.4	10.9	19.8	8.7
GC content of alpha-terpineol (%)		4.0	3.9	5.6	8.9	4.8
Alpha-terpinol selectivity (%)		58.8	53.0	52.6	46.0	56.4

Note: The mass ratio of alpha-pinene, water, AHA, and phosphoric acid was 1:1:0.1:0.05 at 70 °C for 24 h.

**Table 4:** Influence of phosphoric acid on the hydration reaction when acetic acid was used as the auxiliary agent.

	Catalyst	Citric Acid	L-(+)-Tartaric Acid	DL-Mandelic	L-(+)-Lactic Acid	Glycolic Acid
Parmeter						
Alpha-pinene conversion (%)		95.2	97.1	94.8	98	99.5
GC content of alpha-terpineol (%)		46.9	42.1	38.8	40.5	39.5
Alpha-terpinol selectivity (%)		48.1	44.2	40.1	42.2	40.5

Note: the mass ratio of alpha-pinene, water, acetic acid, AHA, and phosphoric acid was 1:1:2.5:0.05:0.05 at 70 °C for 15 h.

**Table 5:** Results and analysis of the orthogonal experiment.

No.	A	B	C	D	E	F	Alpha-Pinene Conversion (%)	Alpha-Tterpinol Selectivity (%)
1	1	2	1	3	3	2	77.0	45.9
2	2	2	3	3	1	1	47.9	43.9
3	1	1	1	1	1	1	16.9	38.3
4	3	3	1	1	2	2	74.5	46.4
5	2	3	2	1	1	3	57.1	45.8
6	3	2	1	2	1	3	99.5	41.3
7	2	3	1	2	3	1	54.8	48.4
8	1	2	3	1	2	3	69.9	48.1
9	3	2	2	1	3	1	71.7	41.6
10	1	1	2	2	3	3	99.8	32.6
11	1	3	2	3	2	1	20.6	51.9
12	3	3	3	3	3	3	99.9	24.9
13	1	3	3	2	1	2	15.2	51.0
14	2	2	2	2	2	2	86.3	46.4
15	3	1	3	2	2	1	95.2	35.8
16	2	1	1	3	2	3	100	37.8
17	3	1	2	3	1	2	96.3	36.5
18	2	1	3	1	3	2	74.3	41.4
Alpha-pinene conversion	K1	299.4	452.3	422.8	441.8	477.7	423.7	
	K2	420.4	482.6	307.3	364.5	333.0	307.3	
	K3	537.3	322.2	431.8	450.9	446.5	456.3	
	R	79.3	53.5	41.5	28.8	48.2	49.7	
Alpha-tterpinol selectivity	K1	267.9	267.2	258.2	241.0	234.9	267.7	
	K2	263.8	222.5	209.3	261.7	256.9	260.0	
	K3	226.7	268.6	254.9	255.6	266.5	182.6	
	R	13.7	15.3	16.3	6.9	10.5	28.4	

**Table 6:** Reaction results for the different initiators.

Product	Materials			
	Terpineyl Acetate		Alpha-Pinene	
	A	B	C	D
	Presence of Acetic Acid	Absence of Acetic Acid	Water	Trace Water
Camphene	-	-	4.5	-
Limonene	15.5	1.1	12.4	11.1
Iso-terpinene	18.8	1.2	17.2	9.2
Fenchyl alcohol	0.5	-	2.1	0.1
β-Terpineol	0.9	-	0.4	-
Isoborneol	-	-	0.2	-
Borneol	-	-	0.6	0.1
4-Terpineol	1.7	0.2	2.6	1.3
Alpha-terpineol	43.7	8.8	46.9	2.3
γ-Terpineol	1.5	0.2	2.3	2.5
Bornyl acetate	-	-	2.2	0.4
Terpineyl acetate	3.3	86.7	3.6	14.1

**Table 7:** Effects of inorganic acids on hydration of alpha-pinene.

	Catalyst	Sulfuric Acid, 0.2%	Citric Acid and Sulfuric Acid	Graphene Oxide, 2%	Citric Acid and Graphene Oxide	Phosphoric Acid, 5%	Citric Acid and Phosphoric Acid
Parmeter							
Alpha-pinene conversion (%)		99.6	98.4	84.7	91.7	89.7	95.2
GC content of alpha-terpineol (%)		38.1	41.8	37.0	39.8	37.2	46.9
Alpha-tterpinol selectivity (%)		39.0	43.3	44.5	44.3	42.3	48.1

Note: The mass ratio of alpha-pinene, water, and acetic acid was 1:1:2.5, at 70 °C for 15 h. The concentrations of sulfuric acid, graphene oxide, phosphoric acid, and citric acid to alpha-pinene were 0.2%, 2%, 5%, and 5%, respectively.

**Table 8:** Citric acid, trichloroacetic acid, and phosphoric acid formed the composite catalysts.

	Catalyst	Citric Acid and Phosphoric Acid	Trichloroacetic Acid and Phosphoric Acid
Parmeter			
Alpha-pinene conversion (%)		97.8	91.2
GC content of alpha-terpineol (%)		46.9	28.1
Alpha-tterpinol selectivity (%)		48.1	30.9

Note: The mass ratio of alpha-pinene, acetic acid, water, citric acid (TCA), and phosphoric acid was 1:2.5:1:0.1:0.05 at 70 °C for 15 h.

**Table 9:** Orthogonal experiment design scheme.

Level	Factors					
	A	B	C	D	E	F
	Mass Ratio of Acetic Acid to Alpha-Pinene (%)	Mass Ratio of Water to Alpha-Pinene (%)	Mass Ratio of Citric Acid to Alpha-Pinene (%)	Mass Ratio of Phosphoric Acid to Alpha-Pinene (%)	Time (h)	Temperature (°C)
1	100	50	5	2	5	60
2	200	100	10	5	10	70
3	300	150	15	8	15	80

## Data Availability

All relevant data are included in the article.

## Sample Availability

Samples of the compounds are not available from the authors.
